# Study on Cross-Coupled-Based Sensing Circuits for Nonvolatile Flip-Flops Operating in Near/Subthreshold Voltage Region

**DOI:** 10.3390/mi12101177

**Published:** 2021-09-29

**Authors:** Taehui Na

**Affiliations:** Department of Electronics Engineering, Incheon National University, Incheon 22012, Korea; taehui.na@inu.ac.kr; Tel.: +82-32-835-8452

**Keywords:** low voltage, nonvolatile flip-flop, sensing circuit

## Abstract

To date, most studies focus on complex designs to realize offset cancelation characteristics in nonvolatile flip-flops (NV-FFs). However, complex designs using switches are ineffective for offset cancelation in the near/subthreshold voltage region because switches become critical contributors to the offset voltage. To address this problem, this paper proposes a novel cross-coupled NMOS-based sensing circuit (CCN-SC) capable of improving the restore yield, based on the concept that the simplest is the best, of an NV-FF operating in the near/subthreshold voltage region. Measurement results using a 65 nm test chip demonstrate that with the proposed CCN-SC, the restore yield is increased by more than 25 times at a supply voltage of 0.35 V, compared to that with a cross-coupled inverter-based SC, at the cost of 18× higher power consumption.

## 1. Introduction

The nonvolatile flip-flop (NV-FF) is regarded as a potential substitute for the conventional volatile FF [1,2,3,4] because of advantages such as zero standby power consumption in the standby mode (power saving), instant-ON from power-down conditions (user-experience improvement and power saving), instant-OFF to the standby mode (power-saving and nonrequirement of external NV memory), and prevention of sudden power failure (reliability improvement). Among the various NV-FF implementations, spin-transfer-torque magnetic tunnel junction (STT-MTJ)-based NV-FFs are considered promising due to their characteristics, including nonvolatility, high endurance, long retention time, CMOS compatibility, scalability, and nil area overhead because of stacking above a MOS transistor [5,6,7,8]. An STT-MTJ-based NV-FF has four operational modes: In the normal FF mode, it functions as a conventional volatile FF; in the backup mode, it stores computing data in the STT-MTJs; in the standby mode, the system powers off completely, resulting in zero standby power; in the restore mode, the stored data from the STT-MTJs are restored to the FF core. It is worth noting that NV-FF design should not degrade the performance of the normal FF mode because normal FF mode operation is the predominant operation in an NV system, whereas restore and backup mode operations occur infrequently in Internet-of-Things (IoT) applications.

Recently, it was suggested that offset-cancellation sensing-circuit-based NV-FFs are insensitive to the offset voltage caused by process variation, improving the restore yield [5,6]. However, these NV-FFs are only valid in the super-threshold voltage region, and are very sensitive to process variations in the near/subthreshold voltage region, because multiple switches for offset-cancellation operation become significant contributors to the offset voltage.

In this study, we investigate two cross-coupled-based sensing circuits for an NV-FF operating in the near/subthreshold voltage region (<0.4 V): a popular cross-coupled inverter-based sensing circuit (CCI-SC) [7,8], and the proposed cross-coupled NMOS-based sensing circuit (CCN-SC). We demonstrate that the CCN-SC achieves better restore yield in the near/subthreshold voltage region on the notion that the simplest is the best. For testing the restore yield, a test IC containing 8 × 8 CCI-SC and CCN-SC arrays is fabricated using 65 nm CMOS technology. The simulation/measurement results show that, compared to the CCI-SC, the proposed CCN-SC has more than 25× restore yield at a supply voltage (V_DD_) of 0.35 V.

## 2. State-of-the-Art NV-FFs

**Figure 1:**
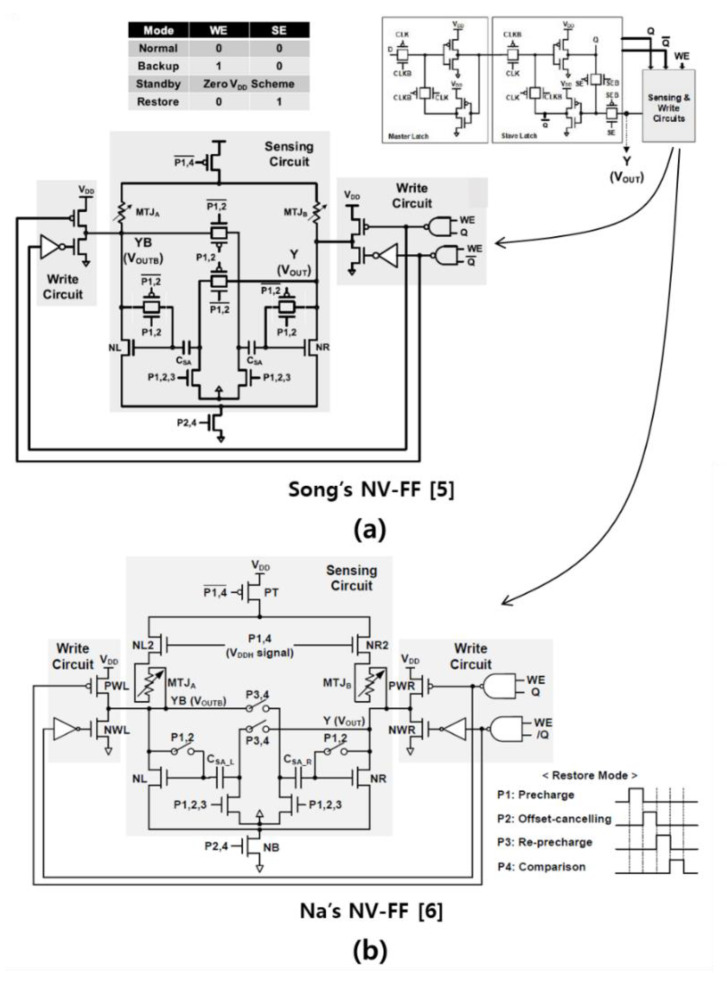
Circuit diagrams of state-of-the-art NV-FFs: (**a**) Song’s NV-FF [5]; (**b**) Na’s NV-FF [6]. Figure 1a is reproduced/adapted with permission from ref [5].

**Figure 2:**
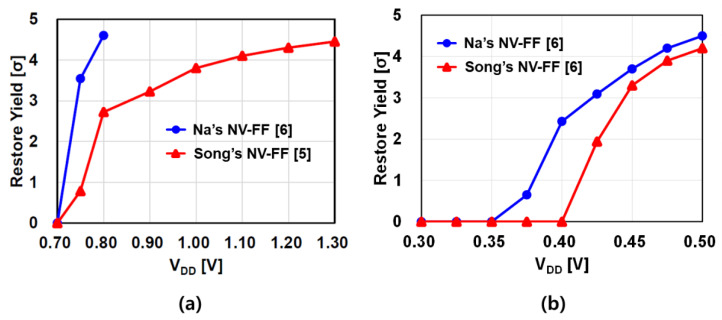
Simulated restore yield of the state-of-the-art NV-FFs according to V_DD_. (**a**) Typical V_th_ devices are used for PT, NL, NR, and NB. For all the other transistors, low-V_th_ devices are used. In this simulation, MTJ_A_ = 3 kΩ, MTJ_B_ = 6 kΩ, each phase time = 20 ns, C_SA_ = 20 fF, C_CP_ = 10 fF, W_PT_ = 2 μm, W_NB_ = W_NL_ = W_NR_ = W_NL2_ = W_NR2_ = 1 μm, W_switch_ = 0.21 μm, and the minimum length = 0.06 μm for all the transistors. (**b**) Extremely large devices (10 times larger than the condition in Figure 2a) and low-V_th_ devices are used for all the transistors. In this simulation, MTJ_A_ = 3 kΩ, MTJ_B_ = 6 kΩ, each phase time = 200 ns, C_SA_ = 200 fF, C_CP_ = 100 fF, W_PT_ = 20 μm, W_NB_ = W_NL_ = W_NR_ = W_NL2_ = W_NR2_ = 10 μm, W_switch_ = 2.1 μm, and the minimum length = 0.06 μm for all the transistors.

Figure 1 shows the circuit diagrams of two state-of-the-art NV-FFs [5,6]. To independently optimize the SC and flip-flop core, the two NV-FFs are based on the separated latch and sensing circuit structure [9,10]. Even though these two NV-FFs have offset cancelation characteristics, offset cancelation becomes ineffective as V_DD_ decreases. This is because not only the main transistors (NL and NR) for amplification but also the other transistors acting as switches have a significant impact on the restore yield. Figure 2a indicates that the restore yield of the two state-of-the-art NV-FFs become zero when V_DD_ is 0.7 V. Even if extremely large devices and low threshold voltage (V_th_) devices are used for all the transistors, the restore yield becomes zero when V_DD_ is 0.35 V as depicted in Figure 2b. Note that the pMOSCAP size must be impractically large (200 μm/0.1 μm (W/L)) to have a capacitance of 200 fF. This figure clearly indicates that even if the NV-FF size is impractically increased, the target restore yield of 4σ cannot be achieved when V_DD_ < 0.4 V.

How can V_DD_ be further decreased to reduce the overall power consumption of IoT/mobile devices? All digital units such as inverters, NAND, and NOR gates can operate correctly even at V_DD_ = 0.4 V or less unless the performance is not a matter. Thus, if the V_DD_ of the NV-FF is reduced, the overall V_DD_ for IoT/mobile devices can be reduced, resulting in an ultra-low-power design.

## 3. Proposed CCN-SC

**Figure 3:**
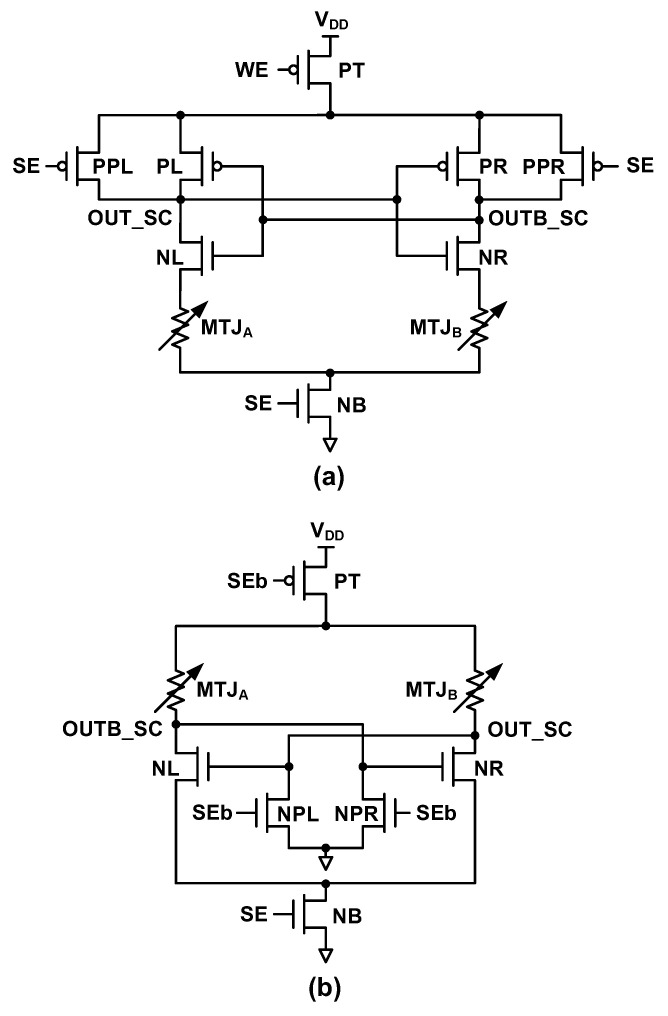
Circuit diagrams of cross-coupled-based SCs for NV-FFs: (**a**) CCI-SC [7,8]; (**b**) Proposed CCN-SC.

As previously mentioned, the state-of-the-art NV-FFs with complex operations are highly ineffective for offset cancelation if V_DD_ is lower than the near-threshold voltage region. Interestingly, a simple circuit that does not include complex offset cancelation could be better for restoring operations when V_DD_ is in the near/subthreshold region as per the simplest is the best concept (there are fewer contributors to the offset voltage). Figure 3a shows the circuit diagram of the CCI-SC [7,8]. The circuit of the proposed CCN-SC (Figure 3b) is simpler than that of the CCI-SC because the total number of transistors is reduced from eight to six, and the number of critical transistors influencing the restore yield is reduced from six (PT, PL, PR, NL, NR, NB) to four (PT, NL, NR, NB).

The restore mode operation of the CCI-SC includes the following two phases: precharge and comparison. In the precharge phase, the write enable (WE) and sense enable (SE) signals are zero. Thus, both OUT_SC and OUTB_SC are precharged to V_DD_. In the comparison phase, WE remains zero but SE becomes unity. The stored data in the MTJs are first compared based on the difference in resistance between MTJ_A_ (R_MTJ_A_) and MTJ_B_ (R_MTJ_B_) and are then amplified by the positive feedback of the CCI (PL, NL, PR, and NR). If R_MTJ_A_ is lesser than R_MTJ_B_, OUT_SC is discharged more rapidly than OUTB_SC. Further, using positive feedback, V_OUT_SC_ and V_OUTB_SC_ are amplified to rail-to-rail voltages (GND and V_DD_, respectively). The restore mode operation of the CCN-SC is almost the same as that of the CCI-SC except for the GND precharge of OUT_SC and OUTB_SC in the precharge phase, more rapid charging of OUTB_SC than OUT_SC in the comparison phase if R_MTJ_A_ is lesser than R_MTJ_B_, and finally, the non-rail-to-rail voltages V_OUT_SC_ and V_OUTB_SC_ because of the intrinsic nature of the CCN structure. These non-rail-to-rail voltages may necessitate additional cross-coupled PMOS latch circuits or latch-type sense amplifiers (SAs) in the CCN-SC if an inverter is insufficient for converting the non-rail-to-rail voltages to rail-to-rail ones. Note that the write circuit shown in Figure 1 can be used for the proposed CCN-SC.

## 4. Measurement/Simulation Results

**Figure 4:**
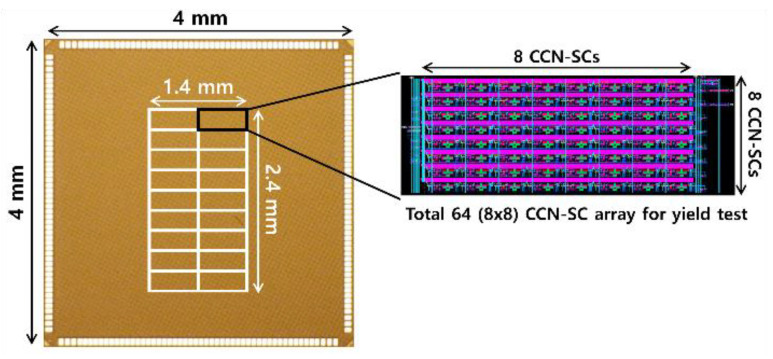
Die image of the test chip implemented using 65 nm CMOS technology.

Figure 4 displays the die photo and structure of the test chip implemented using 65 nm CMOS technology. The structure includes nine 8 × 8 CCI-SC arrays and 8 × 8 CCN-SC arrays, each, with different sizes and resistances for yield testing. Diffusion resistors are used for the MTJ resistance [11]. To compare the restore yield under the same conditions, a voltage-latched SA with double switches and transmission gate access transistors (DSTA-VLSA) [11,12] is used in the CCI-SC as well as CCN-SC. The low MTJ resistance value of 3–5 kΩ is based on [13,14,15,16,17].

**Figure 5:**
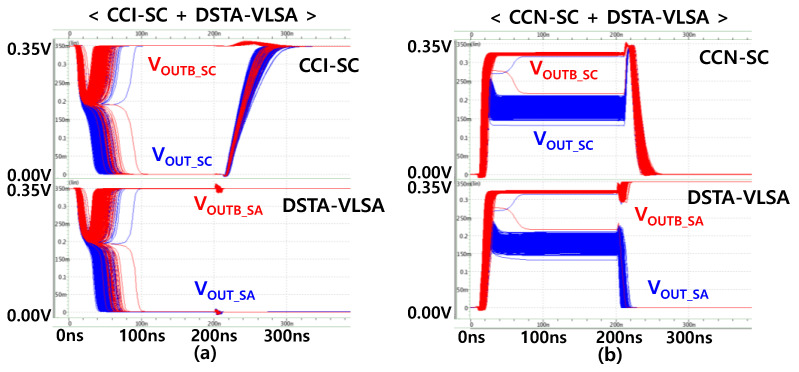
Simulated transient responses. In this simulation, V_DD_ = 0.35 V, MTJ_A_ = 5 kΩ, MTJ_B_ = 6 kΩ, and the critical transistor (PT, PL, PR, NL, NR, NB in CCI-SC, and PT, NL, NR, NB in CCN-SC) width = 128 μm. 1000 sets of Monte Carlo simulations are performed. (**a**) CCI-SC + DSTA-VLSA. (**b**) CCN-SC + DSTA-VLSA.

Figure 5 shows the simulated transient responses of the CCI-SC + DSTA-VLSA and CCN-SC + DSTA-VLSA. V_OUT_SA_ and V_OUTB_SA_ are the output voltages of the DSTA-VLSA. The V_DD_ precharge of CCI-SC, GND precharge of CCN-SC, and the non-rail-to-rail output voltages of CCN-SC are depicted. In addition, it is indicated that the CCN-SC (only one failed sample among 1000 simulations) has a better restore yield than the CCI-SC (more than 10 failed samples).

**Figure 6:**
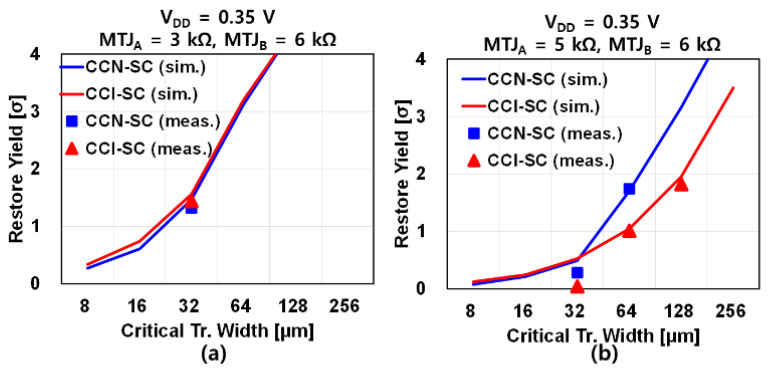
Simulated/measured restore yield according to the critical transistor width. A fixed width of 4 μm is used for the precharge transistors (PPL, PPR, NPL, NPR) and DSTA-VLSA transistors. A length of 0.06 μm is used for all the transistors. (**a**) When V_DD_ = 0.35 V, MTJ_A_ = 3 kΩ, MTJ_B_ = 6 kΩ. (**b**) When V_DD_ = 0.35 V, MTJ_A_ = 5 kΩ, MTJ_B_ = 6 kΩ.

Figure 6 shows the simulated and measured restore yield of the CCI-SC and CCN-SC according to the critical transistor width at V_DD_ = 0.35 V. Five test chips were used for the measurements. Compared to Figure 2b, which shows that the state-of-the-art NV-FFs have zero restore yield at V_DD_ = 0.35 V even if the size is impractically large, Figure 6a with the same MTJ condition clearly shows that the restore yield of both CCI-SC and CCN-SC can be positive and increase with the critical transistor width. Even though the restore yield of CCI-SC and CCN-SC are almost the same when the resistance difference between MTJ_A_ and MTJ_B_ is 3 kΩ (Figure 6a), Figure 6b demonstrates that when the resistance difference decreases to 1 kΩ considering MTJ variation, the restore yield of the CCN-SC decreases slightly, whereas that of the CCI-SC decreases drastically. This is because, in the CCI-SC, not only the NL/NR V_th_ mismatch but also the PL/PR V_th_ mismatch degrades the restore yield, whereas, in the CCN-SC, only the NL/NR V_th_ mismatch degrades the restore yield. In addition, because of the lower IR drop from V_DD_ to GND in the CCN-SC (due to the absence of a PL/PR transistor), the effective V_DD_ is higher. Therefore, the CCN-SC has a better restore yield than the CCI-SC.

Quantitatively, when the resistance difference between MTJ_A_ and MTJ_B_ is 1 kΩ, and the critical transistor width is 64 μm (128 μm), the measured restore yield of the CCI-SC and CCN-SC are 1.01σ (1.82σ) and 1.74σ (>3σ, no failed samples), respectively. This corresponds to restore failure rates of 15.62% (3.44%) and 4.09% (<0.13%), respectively. Thus, by employing the proposed CCN-SC in an NV-FF, the restore yield can be improved by 3.8x (>25×). It should be noted that when the critical transistor width is the same, the layout area of the CCN-SC is only 2/3 that of the CCI-SC because of the more compact and simpler circuit. Thus, when the restore yield is compared in the iso-area condition, the restore yield difference between the CCI-SC and CCN-SC is expected to increase. Even though the power consumption of the CCN-SC is 18 times higher under the condition shown in Figure 5 because of the DC current caused by the non-rail-to-rail output voltages, decreasing V_DD_ can further reduce the system power. In addition, the restore mode power consumption of the NV-FF can be ignored because restore mode operation occurs infrequently in IoT applications.

## 5. Conclusions

This paper proposed a novel CCN-SC that can improve the restore yield, based on the concept that the simplest is the best, of an NV-FF operating in the near/subthreshold voltage region. Experimental results using a fabricated 65 nm test chip as well as simulation results proved the effectiveness of the proposed CCN-SC with which more than 25 times improvement in the restore yield was achieved, compared to the CCI-SC, at a cost of 18 times higher power consumption.
